# Assessing Fungal Population in Soil Planted with *Cry1Ac* and *CPTI* Transgenic Cotton and Its Conventional Parental Line Using 18S and ITS rDNA Sequences over Four Seasons

**DOI:** 10.3389/fpls.2016.01023

**Published:** 2016-07-12

**Authors:** Xiemin Qi, Biao Liu, Qinxin Song, Bingjie Zou, Ying Bu, Haiping Wu, Li Ding, Guohua Zhou

**Affiliations:** ^1^Department of Pharmacology, Jinling Hospital, State Key Laboratory of Analytical Chemistry for Life Science, School of Medicine, Nanjing UniversityNanjing, China; ^2^Department of Pharmaceutical Analysis, China Pharmaceutical UniversityNanjing, China; ^3^Key Laboratory of Biosafety, Ministry of Environmental Protection of China, Nanjing Institute of Environmental SciencesNanjing, China; ^4^Huadong Research Institute for Medicine and BiotechnicsNanjing, China

**Keywords:** farmland ecosystem, fungal diversity, genetically modified cotton, indicator taxa, pyrosequencing, seasonality

## Abstract

Long-term growth of genetically modified plants (GMPs) has raised concerns regarding their ecological effects. Here, FLX-pyrosequencing of region I (18S) and region II (ITS1, 5.8S, and ITS2) rDNA was used to characterize fungal communities in soil samples after 10-year monoculture of one representative transgenic cotton line (TC-10) and 15-year plantation of various transgenic cotton cultivars (TC-15mix) over four seasons. Soil fungal communities in the rhizosphere of non-transgenic control (CC) were also compared. No notable differences were observed in soil fertility variables among CC, TC-10, and TC-15mix. Within seasons, the different estimations were statistically indistinguishable. There were 411 and 2 067 fungal operational taxonomic units in the two regions, respectively. More than 75% of fungal taxa were stable in both CC and TC except for individual taxa with significantly different abundance between TC and CC. Statistical analysis revealed no significant differences between CC and TC-10, while discrimination of separating TC-15mix from CC and TC-10 with 37.86% explained variance in PCoA and a significant difference of Shannon indexes between TC-10 and TC-15mix were observed in region II. As TC-15mix planted with a mixture of transgenic cottons (Zhongmian-29, 30, and 33B) for over 5 years, different genetic modifications may introduce variations in fungal diversity. Further clarification is necessary by detecting the fungal dynamic changes in sites planted in monoculture of various transgenic cottons. Overall, we conclude that monoculture of one representative transgenic cotton cultivar may have no effect on fungal diversity compared with conventional cotton. Furthermore, the choice of amplified region and methodology has potential to affect the outcome of the comparison between GM-crop and its parental line.

## Introduction

Genetically modified plants (GMPs) have an improved quality and higher yield than unmodified plants do. The GMPs that have been developed and marketed currently include transgenic rice, transgenic cotton and transgenic corn. The global commercial cultivation of transgenic crops has increased from 1.7 million hectares in 1996 to 170 million hectares in 2012 in 28 countries (Clive, 2013), including US, Brazil, and Australia. In 2006, cotton and maize expressing *Bacillus thuringiensis* proteins were grown on 32.1 million hectares worldwide (Romeis et al., 2006). Because cotton is an economically important crop worldwide, a pesticidal property was introduced into cotton by expressing an insect-resistant protein of *B. thuringiensis* into the cotton genome. It was reported that *B. thuringiensis* caused an osmotic imbalance or opening of ion channels which activated cell death. It is specifically lethal to Lepidopteran and coleopteran insects (Melo et al., 2016). The insect resistance was greatly improved in GM cotton, which resulted in high yields of cotton and reduced use of insecticides. The GM cotton expressing Cry1Ab/c has been cultivated commercially for more than a decade in China, and it currently represents 71.5% of the total cotton grown because of its low production costs (Ronald, 2014; Carrière et al., 2015).

Because GMPs were first commercialized in 1994, they were welcomed by farmers and consumers, but there remained strong concern regarding the potential impact of GMPs on the environment. In 2001, the US Environmental Protection Agency (EPA; Washington, DC, USA) reassessed *B. thuringiensis* crops that had been accepted for agricultural use for six years (from 1995 to 2001). Investigations have been conducted to evaluate whether GMPs affect the natural environment, including invasiveness, non-target species, the potential of transgenes to “escape” into the environment, and the development of resistance to transgene-derived proteins (Jin et al., 2014). Although the *B. thuringiensis* protein is effective in controlling certain pests, it is important to examine its effects on non-target organisms in the soil. It has been reported that *B. thuringiensis* plants have little impact on the soil biota, such as earthworms, collembolans, and the general soil microflora (Li et al., 2011, 2012; Fließbach et al., 2012; Li and Liu, 2013), and these plants confer an environmental advantage over that require insecticides. However, it is not clear whether *B. thuringiensis* in root exudates influence soil microbial communities directly or indirectly (Li et al., 2015) and how a mixture of GMPs shape microbial communities. Preliminary research has indicated that the contents of root exudates differ significantly between GM cotton and conventional cotton. Fungi are directly exposed to GMP roots, and thus, strong feedback due to the interactions between fungi and GMPs would occur, influencing production and vegetation dynamics. In 1929, fungal pathogens caused a 10% loss of cereal yield according to German authorities (Fisher et al., 2012). In Indian, fungal diseases are regarded as the most important factor contributing to yield losses. The techniques of genetic transformation to develop transgenic resistant to fungal diseases have been even developed (Denning and Bromley, 2015). Therefore, investigation of fungal diversity should be welcomed for illuminating the interaction between GMPs and fungi. The populations of cultivable fungi have increased in some parts of GMPs (Hawes et al., 2012; Li et al., 2015), and therefore, the increase in verticillium wilt and fusarium wilt in *B. thuringiensis* cotton may be related to fungi. To validate this hypothesis, a traditional technique is the use of AMF (arbuscular mycorrhizal fungi), which are considered to be an excellent indicator of the possible ecological impact of GMPs (Meyer et al., 2013; Birkett and Pickett, 2014). However, more diverse information may be lost when AMF are selected as the only target. In addition, controlled laboratory conditions may not represent the actual environment, and thus, research based on AMF alone is insufficient. To better understand the linkage between fungal communities and GMPs, it is necessary to investigate the composition of fungal communities in soil planted with conventional and *B. thuringiensis* cotton.

Several methods may be used to detect fungal diversity. Although DGGE, RFLP, ARDRA, and clone sequencing methods (Anderson et al., 2014; Buscardo et al., 2015; Hu et al., 2015) permit the detection of fungal diversity, the communities identified using these methods appeared to be less taxonomically rich, and changes in the relative abundance of species were easy to overlook based on plate counts of cultivable organisms (Epstein, 2013). Using the clone sequencing method, it was difficult to determine how many clones were required to represent the diversity of a single sample. The best technique is next-generation DNA sequencing (NGS), which allows the sequencing of millions of DNA fragments in parallel. As pyrosequencing-based NGS provides the power to sequence a read length of more than 500 bp (Hol et al., 2015; Hossain et al., 2015; Kazeeroni and Al-Sadi, 2016), we employed this sequencing tool for the comprehensive investigation of the microbial community composition in soil planted with conventional cotton for 15 years (CC), monoculture of one representative *B. thuringiensis* cotton line for 10 years (TC-10), and a mixture of transgenic cotton cultivars for 15 years (TC-15mix).

## Materials and Methods

### Sample Collection and Physicochemical Analyses

This study was conducted in a cotton farm in Baibi town, Anyang, Henan Province, China, which belonged to the Cotton Research Institute (CRI) of the Chinese Academy of Agricultural Sciences (CAAS). The experimental field had a temperate continental monsoon climate with a mean annual rainfall of 556.8 mm and mean annual sunshine hours and temperature of 2228.8 h and 14.1°C, respectively. The soil samples were collected from three experimental fields planted with conventional cotton for 15 years (CC), monoculture of one representative transgenic *B. thuringiensis* cotton line for 10 years (TC-10), and a mixture of transgenic cotton cultivars for 15 years (TC-15mix, **Table 1**) in the seeding stage (S, 26 April), bud stage (B, 13 July), blooming stage (Bl, 22 August) and boll opening stage (Bo, 17 October) in 2011. Each field was separated by a distance of 100 m in this farm to achieve equivalent environments. Fifteen meters were permitted at both ends of every treatment to eliminate marginal field effects on soil sampling. Each type of treatment field was established in quintuplicate (S and B stages) or triplicate (Bl and Bo stages), and each replicate plot was 0.4 hectares. After the weeds and leaves were removed from the surface, soil sub-samples were collected between 0 and 20 cm deep using a soil auger with a diameter of 4 cm. Three replicate soil sub-samples per plot were collected according to the checkerboard method (Committee of handbook of soil fauna research methods, 1998) using a sterile tube and immediately placed on ice for transport to the laboratory. Then the three replicate sub-samples were pooled together and mixed adequately for subsequent DNA extraction and elemental analysis (Supplementary Table S1). The samples were maintained at -80°C for elemental analysis within 7 days according to the National Standard of the People’s Republic of China. DNA sequencing of each sample used for elemental analysis was performed.

**Table 1:** Planting information for three cotton fields.

Treatment	Cotton variety	Foreign gene expressed	Planting time
CC	Zhongmian-35	-	1997 up to now
TC-10	Zhongmian-41	Cry 1Ac and CPTI	2002 up to now
TC-15mix	Mixture of Zhongmian-29, 30, and 33B	Cry 1A	1997-2001
	Zhongmian-41	Cry 1Ac and CPTI	2002 up to now

### DNA Extraction and Pyrosequencing

A total of 48 samples (16 each from one type of treatment field) were analyzed in this study. Genomic DNA extracted from 0.25 g soil sample was prepared for pyrosequencing using the MO BIO Power Soil DNA Extraction kit according to the manufacturer’s protocol (MO BIO Laboratories, Carlsbad, CA, USA). An additional step was included before the final elution step in which the DNA was incubated at 65 °C for 5 min. Finally, the DNA was eluted and collected in 50 μL C6 buffer (provided in the kit). The region I primers (18S) contained the Roche Life Science A or B Titanium sequencing adapter (italicized) followed immediately by a unique 10-base barcode sequence (BBBBBBBBBB) and finally the 3′ end of the primer: R1-F 5′-*CAT CTC ATC CCT GCG TGT CTC CGA CTC AG* BBB BBB BBB BGA TAC CGT CGT AGT CT-3′ (FF700) and R1-R 5′-*CCT ATC CCC TGT GTG CCT TGG CAG TCT CAG* AGC CAT TCA ATC GGT AGT-3′ (FR1) (Vainio and Hantula, 2000). The region II (ITS1, 5.8S and ITS2) primers contained the Roche Life Science A or B Titanium sequencing adapter (italicized) followed immediately by a unique 10-base barcode sequence (BBBBBBBBBB) and finally the 3′ end of the primer: R2-F 5′-*CAT CTC ATC CCT GCG TGT CTC CGA CTC AG*B BBB BBB BBB GAG GCA ATA ACA GGT CTG TGA TGC-3′ (NS7) and R2-R 5′-*CCT ATC CCC TGT GTG CCT TGG CAG TCT CAG* TCC GCA GGT TCA CCT ACG GA-3′ (NS8) (White et al., 1990).

The pyrosequencing PCR mixtures contained 1.25 U of *Taq* polymerase (Takara Biotechnology, Dalian, Liaoning, China), 2.5 μL of 10× PCR buffer supplied by the manufacturer, 0.5 μL of dNTPs (10 mM), 1 μL of 10 μM reverse primer, 1 μL of 10 μM forward primer, 2 μL of DNA template and water up to 25 μL. The amplification was conducted under the following conditions: an initial denaturation at 95°C for 5 min, 35 cycles at 95°C for 20 s, 58°C for 30 s and 72°C for 45 s, and a final extension at 72°C for 7 min. Negative control reactions without template were consistently performed.

The amplicons were visualized using 1.5% (w/v) agarose gels stained with ethidium bromide and purified with AMPure XP Beads (Beckman Coulter, Danvers, MA, USA) according to the manufacturer’s protocol, followed by concentration and size distribution analysis using DNA 1000 chips on an Agilent 2100 Bioanalyzer (Agilent Technologies, Inc., Waldbronn, Germany). Sequencing was conducted on a GS-FLX Titanium pyrosequencer (Roche Life Sciences, Branford, CT, USA) at the Beijing Genomics Institute (Shenzhen, Guangdong, China).

### Microbial Community Analyses via rDNA Gene Sequencing

The sequences were parsed by barcodes using the Mothur software packages (1) to sort sequences with exact matches to the specific 10-bp barcodes into different samples (one unambiguous mismatch to the sample and two mismatches to the PCR primer were permitted), (2) to trim off adapters, barcodes and primers using default parameters, and (3) to remove sequences containing ambiguous ‘N’ homopolymers exceeding 10 bp or with a length shorter than 200 bp (Griffen et al., 2012; Zhang et al., 2012). Denoised sequences were generated by the ‘Shhh.flows’ command in the Mothur platform to remove sequences that were likely due to pyrosequencing errors. All of the sequences were aligned using a NAST-based sequence aligner to a custom reference based on the SILVA alignment. It was ensured that all of the sequences overlapped in the same alignment space by trimming the ends of each sequence such that all of the sequences started and ended at the same alignment coordinates. All of the sequences were then pre-clustered to permit one base difference in one hundred bases to form a more abundant sequence. Chimeric sequences were then identified using UCHIME (http://drive5.com/uchime). All of the sequences were aligned in the Silva alignment using NAST and then classified using the RDP (Ribosomal Database Project) classifier with an RDP confidence threshold of 80% or greater in the Silva database. The clean dataset was clustered into a molecular operational taxonomic unit (OTU) with a 97% identity threshold using the average neighbor clustering algorithm. The control samples used to validate the abundance data were provided. Both regions were treated equivalently.

OTU richness was determined as the number of OTUs present in a sample. Shannon and Chao 1 were calculated using EstimateS based on 100 randomizations with at least 75% of the sequence selected at one time (Caporaso et al., 2010; Colwell et al., 2012). Shannon diversity was exponentially transformed Exp (H). The relationships between OTU richness, Shannon and soil elements (TOC, TN, TP, and TK) were explored by calculating Pearson’s correlation coefficients among each pair of variables. OTU richness and Shannon among CC, TC-10, and TC-15mix were compared using *t*-tests.

The data were normalized as a percent of the total for each taxon per sample, and the taxa were averaged for region I and region II. The cluster analysis was conducted to group the fungal communities from different soil samples based on the generated OTUs using RDP Complete Linkage Clustering from the merged pool of sequences from all of the samples. The names of the fungal sequences from each sample were specifically encoded to identify their sources in the merged sequence pool. The cluster analysis was then conducted using the unweighted-pair group method with arithmetic mean (UPGMA) based on the distance metric of UniFrac calculated by QIIME (Stoll et al., 2014). It was constructed by aligned, representative sequences for OTUs at 3% cut-off level using Weighted UniFrac with the default settings. Then the similarity cut-off levels clustered the soil samples into several main groups.

PCoA (principal coordinate analysis) was conducted based on the OTUs described above. PCoA is a phylogenetically independent method, and weighted and unweighted UniFrac distances were generated from normalized data in a beta-diversity pattern using a QIIME analysis pipeline. The plots were generated based on the weighted and unweighted UniFrac distance metric (Blaser et al., 2013).

To identify any significant differences among the taxonomic groups from the three soil types, the conventional cotton control samples were compared with the transgenic samples using a Mann-Whitney-Wilcoxon signed-rank test (function wilcox_test in the coin package of R). The obtained *P*-values were adjusted based on a Benjamini-Hochberg false discovery rate correction (function p.adjust in the stats package of R) (Griffen et al., 2012).

## Results

### Soil Fertility Variables

TOC (total organic carbon), TN (total nitrogen), TP (total phosphorus), and TK (total potassium) were analyzed. As shown in Supplementary Table S2, no notable differences were observed in the concentrations of TOC, TN, TP, or TK across the fields (48 soil specimens), with calculated averages of 15.27 ± 2.22, 1.221 ± 0.301, 0.780 ± 0.083, and 14.77 ± 1.31 g/kg, respectively. Therefore, the transgenic cottons had no effects on soil fertility, and farming management practices appeared to be almost equivalent among the three fields.

### Composition and Diversity of Fungal Communities in the Soil

Based on the two sets of primers that were used to analyze fungal diversity in the references (White et al., 1990; Vainio and Hantula, 2000), two different regions (designated “I” and “II”) were sequenced for each sample using the barcoded pyrosequencing platform to yield 1 147 486 sequences. Among these sequences, 323 270 (region I) and 199 301 (region II) qualified sequences were generated from 47 (region I) and 48 (region II) samples, respectively. The numbers of qualified sequences per sample were, on average, 6878 ± 2590 (region I) and 4152 ± 1539 (region II), ranging from 2449 to 13497 and from 1176 to 7892, respectively. Of all the qualified sequences, four fungal phyla were identified and the remaining sequencing data were grouped as unclassified fungi and non-fungi (others). The percentages of fungal sequences in the full pyrosequencing dataset were 70% and 61%, respectively, for region I and II. All but 30 and 39% of the sequences could be classified at the domain level (Algae, Alveolata, Amoebozoa, Cryptophyta, Euglenozoa, Metazoa, Viridiplantae) based on the Silva database in region I and region II, respectively, and these sequences also belonged to eukaryotes (Supplementary Tables S2-S5). The percentages of shared taxa in the two regions were 100, 94, 86, 77, and 62% in region I and 100, 77, 60, 40, and 32% in region II, respectively, at the level of the phylum, class, order, family, and genus.

**FIGURE 1:**
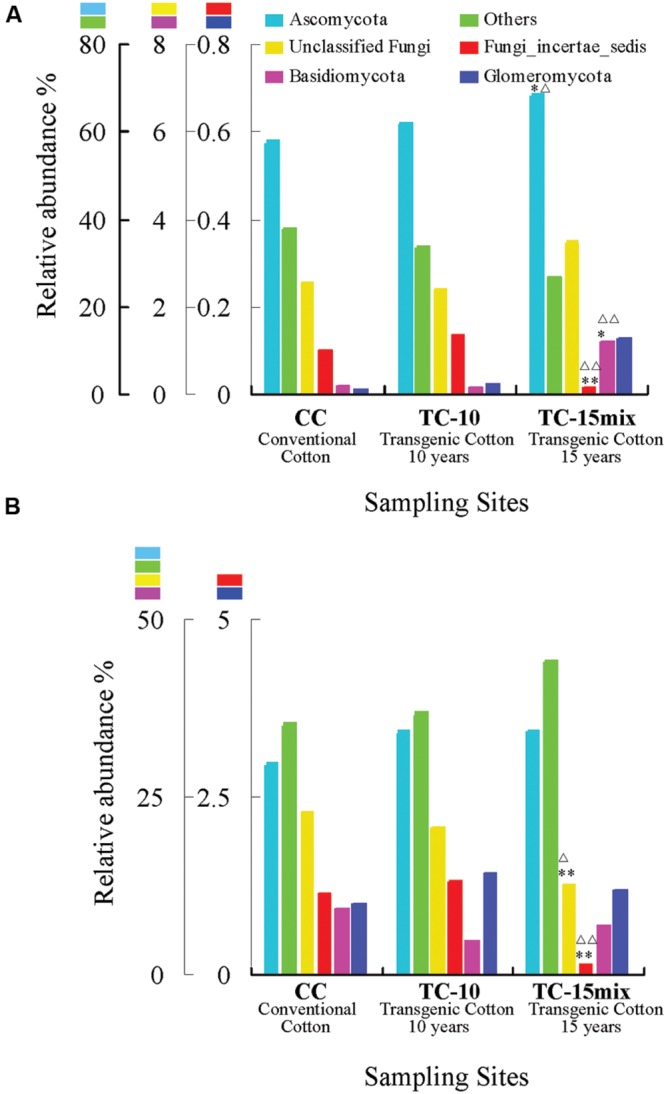
**Relative abundance (%) of fungal phyla in the overall communities of soil samples from different groups (CC, TC-10, and TC-15mix) in region I (A) and region II (B). (A)** Others include Alveolata, Metazoa, Unclassified_eukaryota, Viridiplantae, and Stramenopiles. **(B)** Others include Algae, Alveolata, Amoebozoa, Cryptophyta, Euglenozoa, Metazoa, Viridiplantae, and Unclassified_eukaryota. ^∗^Significant difference with a *P*-value of <0.05 in comparison with CC; ^∗∗^Significant difference with a *P*-value of <0.01 in comparison with CC. ^Δ^Significant difference with a *P*-value of <0.05 in comparison with TC-10; ^ΔΔ^Significant difference with a *P*-value of <0.01 in comparison with TC-10.

**Figure 1** shows the relative abundance of the fungal phyla, unclassified fungi and non-fungi (others) in the overall communities of the soil samples from different groups (CC, TC-10, and TC-15mix) in region I and region II, respectively. In region I (**Figure 1A**), excluding the non-fungi (others), Ascomycota represented the dominant lineage in each group, accounting for 58, 62, and 69% of all sequences in the CC, TC-10, and TC-15mix groups, respectively. Fungi_incertae_sedis accounted for 1.0 and 1.4% of the CC and TC-10 groups, respectively, but decreased to 0.16% in the TC-15mix group. The relative abundance of Basidiomycota and Glomeromycota were 0.12% and 0.13% in TC-15mix, respectively, but reduced to 0.018% and 0.013% in CC, and to 0.014% and 0.022% in TC-10. These data suggested that the relative abundance of Fungi_incertae_sedis, Basidiomycota and Glomeromycota varied largely in the CC, TC-10, and TC-15mix groups and that the relative abundance of the unclassified fungi was nearly equivalent among the samples from the three groups.

In region II (**Figure 1B**), excluding the non-fungi (others), Ascomycota still represented the most dominant lineage in each group, and the relative abundance of Fungi_incertae_sedis also decreased considerably in TC-15mix. In contrast to region I, the relative abundance of Basidiomycota and Glomeromycota from all groups was close to 1%, which was approximately 10 times higher than those in region I. This result indicates that the two regions encompass a different range of species and that each region should be assessed individually in the data analysis.

### Taxa Richness

Approximately 95% (region I) and 69% (region II) of the fungal sequences could be classified at the fungal phylum level (Ascomycota, Basal_fungal_lineages, Basidiomycota and Glomeromycota) using the RDP classifier (80% threshold). A total of 411 (region I) and 2067 (region II) fungal OTUs were observed across all of the sampling locations and seasons. Among the locations, 110 (region I) and 320 (region II) fungal OTUs were present in soil samples from CC and TC, respectively. Although a majority of the OTUs were scattered throughout the samples, their frequencies were much lower. The data indicated that microbial populations tended to have a long tail of less abundant taxa even after rigorous sequence denoising and the exclusion of singletons. Among 35 or more samples, there were 15 (region I) and 22 (region II) OTUs (**Table 2**).

**Table 2:** The most abundant fungal taxa in samples of different sampling sites identified at the OTU level.

OTU rank	Region I					Region II				
	Assigned taxonomy	Frequency				Assigned taxonomy	Frequency			
		CC	TC-10	TC-15mix	Total		CC	TC-10	TC-15mix	Total
1	*Mycosphaerella*	100	100	100	47	*Strobiloscypha*	100	100	100	48
2	*Geomyces*	100	100	100	47	Fungi;other	100	100	100	48
3	*Pezizales*	100	100	100	47	Fungi;other	100	100	100	48
4	*Chaetomium*	100	100	100	47	*Geomyces*	100	100	100	48
5	*Halosphaeriaceae*	100	100	100	47	Fungi;other	100	100	100	48
6	*Verticillium*	100	100	100	47	*Dothideomycetes_incertae_sedis*	100	100	93.8	47
7	*Talaromyces*	93.8	93.8	100	45	Fungi;other	100	93.8	93.8	46
8	*Pleospora*	87.5	100	93.3	44	*Agaricales*	100	87.5	93.8	45
9	*Dikarya;other*	87.5	87.5	100	43	*Kirschsteiniothelia*	93.8	93.8	87.5	44
10	*Pestalosphaeria*	81.3	100	93.3	43	Fungi;other	87.5	93.8	87.5	43
11	*Dikarya;other*	87.5	87.5	86.7	41	*Dothideomycetes*	87.5	93.8	87.5	43
12	*Dothideomycetes*	87.5	81.3	86.7	40	*Agaricomycotina*	87.5	81.3	93.8	42
13	*Pezizomycotina*	87.5	81.3	73.3	38	*Alternaria*	93.8	75.0	87.5	41
14	*Coniosporium*	100	100	20.0	35	*Sordariomycetes*	93.8	87.5	75.0	41
15	*Poitrasia*	81.3	56.3	86.7	35	*Pezizomycotina*	87.5	81.3	87.5	41
16	*Ascomycota*	100	100	6.7	33	*Pleospora*	81.3	75.0	93.8	40
17	*Eurotiomycetidae*	37.5	62.5	100	31	*Pezizales*	100	100	43.8	39
18	*Lecanoromycetes*	62.5	87.5	33.3	29	Fungi;other	93.8	87.5	62.5	39
19	Fungi;other	75.0	50.0	53.3	28	*Pezizomycotina*	100	93.8	50.0	39
20	*Pezizomycotina*	62.5	62.5	53.3	28	*Filobasidiales*	93.8	62.5	81.3	38
21	Fungi;other	56.3	75.0	40.0	27	*Coniosporium*	75.0	81.3	81.3	38
22	*Dikarya;other*	43.8	43.8	86.7	27	Fungi;other	100	81.3	56.3	38
23	*Talaromyces*	62.5	43.8	60.0	26	*Glomeromycetes*	62.5	81.3	87.5	37
24	Fungi;other	62.5	43.8	33.3	22	*Pleosporales*	87.5	75.0	68.8	37
25	*Sarcoscyphaceae*	37.5	37.5	60.0	21	*Pezizales*	81.3	93.8	50.0	36
26	*Ascomycota*	18.8	18.8	60.0	15	*Trichocomaceae*	81.3	87.5	56.3	36
27						*Poitrasia*	93.8	100	25.0	35
28						Dikarya;other	87.5	87.5	43.8	35
29						Fungi;other	62.5	87.5	68.8	35
30						*Agaricomycetes*	68.8	75.0	68.8	34
31						*Basidiomycota*	87.5	81.3	37.5	33
32						*Sordariomycetes*	50.0	62.5	87.5	32
33						Fungi;other	75.0	68.8	50.0	31
34						*Paracoccidioides*	62.5	87.5	37.5	30
35						*Halosphaeriaceae*	68.8	62.5	56.3	30
36						*Agaricomycotina*	62.5	81.3	43.8	30
37						*Paracoccidioides*	37.5	50.0	87.5	28
38						*Glomeromycetes*	56.3	56.3	56.3	27
39						Fungi;other	62.5	43.8	62.5	27
40						*Nectria*	56.3	37.5	68.8	26
41						*Agaricomycetes*	37.5	56.3	68.8	26
42						*Basidiomycota*	68.8	68.8	18.8	25
43						*Sporobolomyces*	87.5	62.5	0.0	24
44						Dikarya;other	43.8	56.3	50.0	24
45						Fungi;other	62.5	50.0	31.3	23
46						Hypocreales	68.8	37.5	37.5	23
47						*Basidiomycota*	50.0	56.3	37.5	23
48						*Dothideomycetidae*	56.3	25.0	56.3	22
49						*Tremellomycetes*	87.5	43.8	6.3	22
50						*Strobiloscypha*	56.3	56.3	18.8	21
51						Fungi;other	87.5	25.0	18.8	21
52						*Strobiloscypha*	75.0	37.5	12.5	20
53						*Dothideomycetes*	18.8	31.3	75.0	20
54						*Pezizomycotina*	37.5	31.3	56.3	20
55						*Basidiomycota*	37.5	37.5	50.0	20
56						*Glomeromycetes*	50.0	43.8	25.0	19
57						*Leptosphaeria_maculans_complex*	50.0	31.3	37.5	19
58						*Basidiomycota*	62.5	43.8	6.3	18
59						Dikarya;other	62.5	31.3	6.3	16
60						Fungi;other	31.3	50.0	18.8	16
61						*Agaricomycetes*	43.8	50.0	6.3	16
62						Dikarya;other	50.0	25.0	25.0	16

The species diversity and combination of homogeneity and richness were reflected by the Chao 1, OTU richness and Shannon. Within seasons, the different estimations were statistically indistinguishable (**Supplementary Figure S1A**), and there were no significant differences among the separate fields (**Supplementary Figure S1B**, *P* > 0.05). The *t*-test also showed both OTU richness and Shannon had no significant difference between CC and TC-10, except for Shannon index between TC-10 and TC-15mix in region II (Supplementary Table S6). Within regions, region II accumulated OTUs at a higher rate across both sampling sites and seasons. Pearson’s correlation coefficients showed that no elements were significantly correlated with diversity indexes (Supplementary Table S7).

### Taxonomic Coverage and Indicator Taxa

**FIGURE 2:**
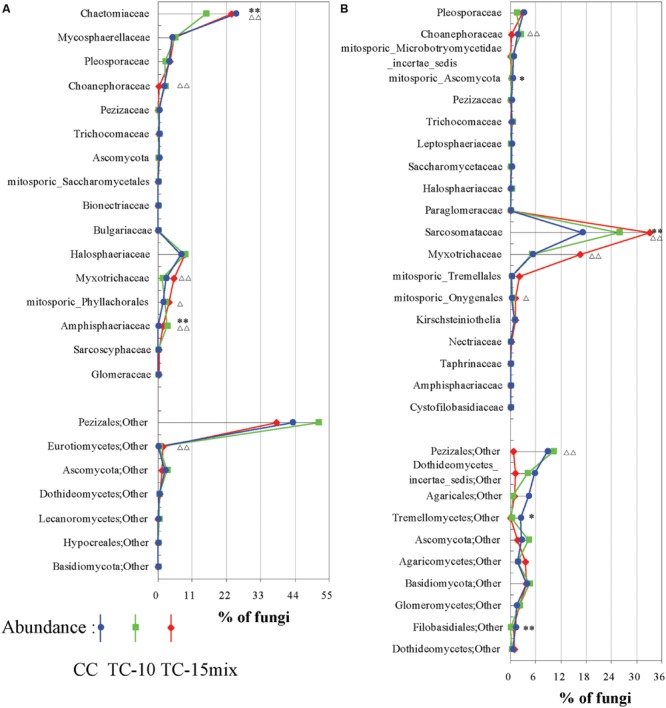
**Differences among CC, TC-10, and TC-15mix at the family level. (A)** Region I; **(B)** Region II. The graphs show families with differences ≥0.03% and abundances ≥0.03% in the CC and TC samples. Some of the families could not be divided into lower levels and were represented by others according to their respective orders. ^∗^Significant difference with a *P*-value of <0.05 between CC and TC-10; ^∗∗^Significant difference with a *P*-value of <0.01 between CC and TC-10; ^Δ^Significant difference with a *P*-value of <0.05 between CC-10 and TC-15mix; ^ΔΔ^Significant difference with a *P*-value of <0.01 between CC-10 and TC-15mix.

In region I, 64% of the highly abundant fungal taxa shown in **Table 2** were identified at different sampling sites (CC-soil, TC-10-soil, and TC-15mix-soil), and these taxa were present in more than half of the samples. However, in region II, there were 54, 47, and 40 taxa in the CC-soil, TC-10-soil, and TC-15mix-soil, respectively, and each taxon was present in more than half of the samples from the same sites. In total, 19 orders of fungi encompassing distinct evolutionary lineages and a diversity of morphologies were discovered (**Table 3**). Based on the two regions, the percentages of taxa shared in CC and TC were 100% and 100%, 100% and 86%, 89% and 81%, 91% and 80%, and 84% and 76% in region I and region II at the level of the phylum, class, order, family and genus, respectively. Abundant families of soil fungi in region I and region II were shown in **Figures 2A,B** respectively. Both regions contained only a few fungi that differed in abundance among the soil samples from CC, TC-10 and TC-15mix at the level of the family. For example, in region II, Sarcosomataceae, Myxotrichaceae and mitosporic_Tremellale were highly abundant in the soil samples from TC-15mix, while Pleosporaceae and Choanephoraceae represented the majority in CC.

**Table 3:** Classes of fungi and their representation.

	Region I						Region II					
	Class	Number of OTUs	Description	CC	TC-10	TC-15mix	Class	Number of OTUs	Description	CC	TC-10	TC-15mix
1	Sordariomycetes	66	Plant pathogens and saprobes	16	16	15	Pezizomycetes	222	Mushrooms and molds	16	16	16
2	Pezizomycetes	40	Mushrooms and molds	16	16	15	Dothideomycetes	162	Molds	16	16	16
3	Dothideomycetes	26	Molds	16	16	15	Agaricomycetes	150	Mushrooms and polypores	16	16	16
4	Ascomycota	21	Mushrooms and polypores	14	9	13	Glomeromycetes	146	Plant root biotrophs	16	16	16
5	Eurotiomycetes	13	Molds	16	16	15	Leotiomycetes	95	Plant pathogens and saprobes	16	16	16
6	Glomeromycetes	12	Plant root biotrophs	5	6	8	Tremellomycetes	63	Yeasts	16	13	14
7	mitosporic_Ascomycota	8	Molds	1	1	1	Sordariomycetes	59	Plant pathogens and saprobes	16	16	16
8	Mucorales	7	Molds	16	16	6	Eurotiomycetes	37	Molds	16	14	16
9	Agaricomycetes	5	Mushrooms and polypores	1	3	4	Basal_fungal_lineages	20	Molds	16	16	8
10	Saccharomycetes	4	Yeasts	7	2	1	Microbotryomycetes	12	Yeasts	14	13	6
11	Lecanoromycetes	3	Lichenized fungi	11	14	5	mitosporic_Ascomycota	10	Molds	15	15	15
12	Leotiomycetes	2	Plant pathogens and saprobes	16	16	15	Taphrinomycetes	7	Plant pathogens	2	3	5
13	Tremellomycetes	2	Yeasts	1	1	1	Cystobasidiomycetes	7	Yeasts	1	0	0
14	Cystobasidiomycetes	1	Yeasts	0	0	1	Lecanoromycetes	6	Lichenized fungi	6	6	5
15	Exobasidiomycetes	1	Plant pathogens	0	0	1	Saccharomycetes	6	Yeasts	7	4	2
16	Fungi;other	85		16	16	15	Exobasidiomycetes	3	Plant pathogens	0	2	3
17	Dikarya;other	74		16	16	15	Dacrymycetes	2	Jelly fungi	2	2	0
18	Pezizomycotina;other	28		16	16	15	Agaricostilbomycetes	1	Molds	0	0	1
19	Basidiomycota;other	13		16	16	15	Pucciniomycetes	1	Molds	1	0	0
20							Fungi;other	403		16	16	16
21							Dikarya;other	284		16	16	16
22							Ascomycota;other	59		16	16	16
23							Pezizomycotina;other	168		16	16	16
24							Basidiomycota;other	79		16	16	16
25							Agaricomycotina;other	64		16	16	16
26							Pucciniomycotina;other	1		16	16	16

*The abundances of OTUs which were identified to class were summed. The representative ecologies for those OTUs were given.*

**FIGURE 3:**
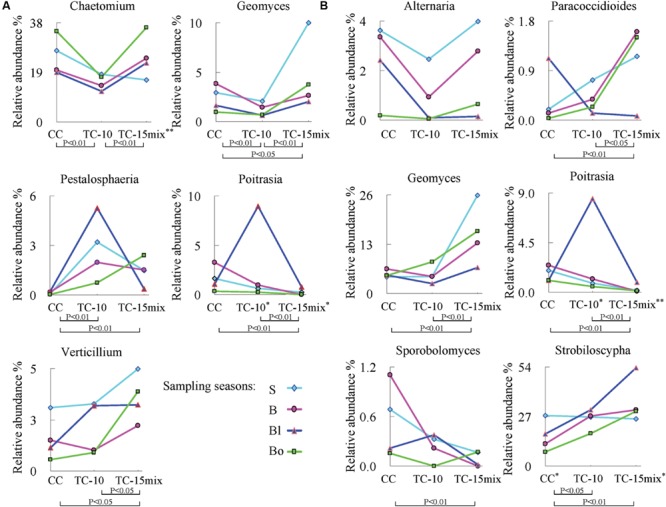
**Significant differences among CC, TC-10, and TC-15mix at the genus level during different seasons (*P* < 0.05). (A)** Region I; **(B)** Region II. The graphs show the levels for genera with frequencies ≥30 in all samples (48 for region I and 47 for region II) and abundances ≥0.01%. S: seeding stage (26 April); B: bud stage (13 July); Bl: blooming stage (22 August); Bo: boll opening stage (17 October) in 2011. ^∗^*P* < 0.05 among the four seasons in the corresponding site. The double asterisks represent significant difference with a *P*-value of <0.01.

The abundance of rare genera specific to CC, TC-10, or TC-15mix was less than 0.065%. Some individual genera with significant differences in abundance among CC, TC-10, and TC-15mix over four seasons (lines with different colors) were shown in **Figure 3**. Both regions contained the genera *Geomyces* and *Poitrasia*, and importantly, each season provided a similar pattern of abundance among CC, TC-10, and TC-15mix. The present study also showed that the abundance of *Alternaria* (region II) and *Chaetomium* (region I) decreased by approximately 30% on average in TC-10 in comparison to CC in all four sampling periods and increased to the same level as CC in TC-15mix. In contrast, for *Poitrasia* (in both regions) and *Petalosphaeria* (region I) at the blooming stage, the abundance increased significantly (approximately 60-fold) in TC-10 compared with CC and decreased to the same level as CC in TC-15mix. Although the abundance of some genera showed significant difference between CC and TC, the proportion of them was less than 10%.

### Similarity Analysis of Samples from the Three Sampling Sites

**FIGURE 4:**
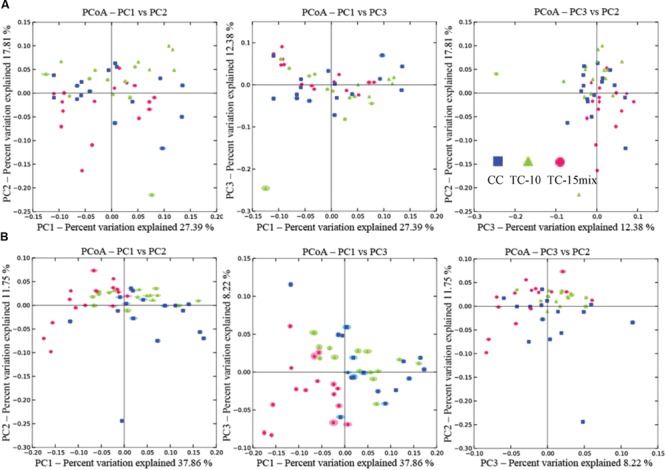
**Principal coordinate analysis of soil samples by weighted UniFrac. (A)** Region I; **(B)** Region II.

The similarity of the 47 samples in region I and 48 samples in region II were evaluated by cluster analysis and principle coordinate analysis (PCoA), respectively. Based on the fungal communities, the soil samples could be clustered into several groups in cluster analysis (**Supplementary Figure S2**). An ambiguous cluster version among samples from CC, TC-10, and TC-15mix was shown in region I. In region II almost all samples from CC separated from those in TC-15mix except for No.33 (branch IV) and 31 (branch II), while CC and TC-10 clustered together. PCoA was also performed to elucidate similarities among different soil samples based on OTUs and exhibited similar observations with cluster analysis. As shown in **Figure 4** (UniFrac at a 3% cutoff), no obvious clusters among the soil samples were observed in region I. PC1 axes separated TC-15mix from CC and TC-10 with 37.86% explained variance in region II.

## Discussion

Although recent research has improved our understanding of microbial communities associated with GMPs (Weinert et al., 2010; Li et al., 2014), studies investigating these pair-wise differences must be examined within a wider context of natural variation for a long planting time (Szénási et al., 2014). This study aimed to elucidate whether transgenic cotton influenced soil fungal communities in natural ecosystem more than 10 years after planting. Moreover, we examined the effects of plant growth stage using two amplified regions to assess the influence of seasonal variation and microbial lineages. Given the current state of our knowledge, no data are available regarding the responses of fungal communities in two amplified regions for the annual cycle of *B. thuringiensis* cotton. We started our investigation with elemental analyses in CC, TC-10, and TC-15mix to determine whether genetic modifications had effects on soil fertility, which in turn may affect the fungal diversity. It was found that genetic modifications did not influence soil fertility, and the elements were not related with taxa richness (Supplementary Tables S1 and S7). Soil fertility analyzed in this study had no interaction with fungal community.

Although many primers are valuable for investigating fungal diversity, unfortunately none of them can capture all diversity in the fungal kingdom with a high specificity (Stukenbrock and McDonald, 2008; Chen et al., 2014; Ene and Bennett, 2014; Schadt and Rosling, 2015). Here, for improved diversity, two primer pairs rather than one were employed to amplify 18S and ITS regions. For instance, *Mycosphaerella, Chaetomium*, and *Verticillium* were dominant in region I, while Strobiloscypha and Dothideomycetes_incertae_sedis were included in region II (**Figure 2**; **Tables 2** and **3**). The inconsistency of the communities between the two regions resulted in some differences in their abundance at the higher level (e.g., unclassified fungi, other and Basidiomycota, as detailed in **Figure 1**). This result was due to much more stratified and complex communities and proportions of unclassified reads in the higher taxonomy. Thus, the different primer combinations may have resulted in variable proportions, especially at the higher taxonomic level. Both primer pairs detected not only independent fungal diversity but also some overlapping taxa. Additionally, the similar spatiotemporal variations in abundance of overlapping taxa validated the accuracy of abundance. For instance, a clear tendency for the rate of change in the relative abundance of Fungi_incertae_sedis in the three fields in region I was in perfect agreement with that in region II, because almost all of the reads for Fungi_incertae_sedis in the two regions were assigned to one taxon, *Poitrasia.* Strong consistencies could also be obtained for Pleosporaceae and Myxotrichaceae at the family level (**Figure 2**). Consequently, the results from two regions yielded a consistent and mutually complementary diversity. Indeed, both regions were necessary to characterize the fungal communities in CC, TC-10, and TC-15mix.

It was found that more than 75% of the highly abundant taxa were stable in soil planted with transgenic and conventional cottons. Ascomycota was the largest phylum of fungi, which is consistent with a previous publication (Hibbett and Taylor, 2013). No significant differences were observed in abundance of phyla between CC and TC-10 except for TC-15mix (**Figure 1**). Our results were consistent with the previous publications, suggesting that monoculture of one transgenic cotton line may have no effect on fungal phylum composition in comparison with conventional cotton (Griffiths et al., 2000; Koch et al., 2015; Zhou et al., 2016). However, a remarkable difference was observed between TC-15mix and groups of CC and TC-10 at phylum level. Researches showed cultivar type influenced microbial community structure (İnceoǧlu et al., 2010; Dias et al., 2012). Even in the context of transgenic crops, different genetic modifications may generate non-desirable phenotypic alterations (Ricroch et al., 2011; Li et al., 2015). Both TC-10 and TC-15mix planted with transgenic cottons, while TC-15mix planted with a mixture of genetically modified cottons for over 5 years and appeared to have a different fungal composition from TC-10, suggesting that the variations introduced by different genetic modifications may be greater than those between transgenic and conventional cottons. Similar observations had been made in both regions. Based on the current data, it is still premature to predict the likelihood of dissimilar fungal diversity in TC-15mix from CC and TC-10. Further clarification is necessary by monitoring the fungal dynamic changes in sites planted in monoculture of various transgenic cottons.

Examinations of the diversity index and community structure data across the samples can help to further illuminate whether transgenic cotton influenced fungal diversity. No discrimination was observed in diversity indexes or fungal communities among the four seasons within one treatment field. The differences in abundance among the four seasons within one field were much smaller than those among the three treatment fields (Supplementary Tables S2-S4; **Supplementary Figure S2**). No significant difference of diversity indexes and no distinct discrimination were observed between CC and TC-10 in both regions (**Figure 4**; Supplementary Table S6; **Figures S1** and **S2**). The results revealed that the structures of communities between CC and TC-10 were quite similar. However, Shannon index in TC-10 was statistically different from that in TC-15mix in region II (Supplementary Table S6). Pearson’s correlation analysis showed differences in diversity indexes were not affected by soil fertility variables. Furthermore, the discriminations between TC-10 and TC-15mix were detected in PCoA and consistent across four seasons (**Figure 4B**). The data showed a similar pattern to that seen at phylum level, and suggested variations among genetic modifications within GMPs may have an effect on microbial diversity, like variations among conventional cultivars (Dias et al., 2012). Thus, fungal diversities were dissimilar between CC and TC-15mix in cluster analysis and PCoA.

Within the amplified regions, as the two pairs of primers targeted different fungal lineages (**Figure 2**; **Tables 2** and **3**), the discrimination of diversity appeared to be more obvious in region II. As no research of microbial diversity associated with planting various transgenic cottons for such a long time has been carried out, further investigation is necessary to clarify this.

Monoculture of one transgenic cotton line had no effect on fungal diversity in comparison with conventional cotton, while fungal population dynamics among CC, TC-10, and TC-15mix were observed. Despite our current understanding of plant-soil community interactions, the mechanism by which *B. thuringiensis* plants drive fungal community dynamics is not well understood. For example, the long-term variation in the plant-specific selection of microbial population in the rhizosphere is unclear. The collected data revealing frequencies of 81% for the dominant taxa showed no obvious tendency among CC, TC-10, and TC-15mix (**Table 2**), while the frequencies of taxa belonging to No. 17 (region I) and 41 (OTU rank order) were highest in TC-15mix and lowest in CC, and the taxa belonging to No.14 and 24 displayed an opposite trend. Similar variations could also be observed in No. 19, 23, 35, and 37 (region II). This indicated such a few taxa may be vulnerable to the plants and soil systems.

The key and indicator taxa are vulnerable to the crop species and may have a crucial role both in clarifying the potential influence of GMPs (Kowalchuk et al., 2003) and in maintaining the soil dynamics. There have been studies on indicators associated with GMPs (Bruinsma et al., 2003; Sarkar et al., 2009; Arango et al., 2014; Cotta et al., 2014), however, the majority of the researches lacked the power to thoroughly estimate rhizosphere microbial diversity and monitor dynamic nature of indicators. In this study, GS-FLX platform gave a better understanding of the dynamic nature of mycorrhizal fungal taxa which were sensitive to disturbances. Myxotrichaceae demonstrated significant differences in abundance between CC and TC (*P* < 0.05), and their cellulolytic ability facilitated the penetration of root cortical cell walls (Dalpé, 1989). In addition, some other families were identified that deserve further investigation (**Figure 2**). Several individual genera with significantly different variations in abundance among CC, TC-10, and TC-15mix were analyzed in **Figure 3**. A similar trendline of variation was observed among CC, TC-10, and TC-15mix within the four seasons. It was suggested that there was a higher abundance of *Verticillium* in TC (*P* = 0.018 for CC vs. TC-15mix, *P* = 0.045 for TC-10 vs. TC-15mix; **Figure 3A**), which is likely to threaten *B. thuringiensis* cotton with wilt diseases (Fradin and Thomma, 2006). This result potentially correlated with the unexpected traits of GMPs reported by several groups, including lower yields and an enhanced susceptibility to pathogens (Pasonen et al., 2004; Zeller et al., 2010). Little information is available for *Geomyces*, except that *Geomyces destructans* is relevant to white-nose syndrome (WNS) in bats (Lorch et al., 2011). *Pestalosphaeria*, a potent plant pathogen, represents the sexual stage of *Pestalotiopsis* (Kang et al., 1999), which causes leaf spots, needle blight and tip blight (Pirone, 1978; Keith et al., 2006). It presents an initial increasing and then decreasing finger period with the exception of Bo. The abundance of *Paracoccidioides* (*P* = 0.006 for CC vs. TC-15mix, *P* = 0.039 for TC-10 vs. TC-15mix) increased from CC to TC-15mix during the sampling periods for S, B, and Bo. The protein TasHyd1 expressed by *Trichoderma* was found to be harmful to plants, participating in plant root attachment and colonization (Viterbo and Chet, 2006). A Class I hydrophobin from one species of *Paracoccidioides* was found to express the protein Pbhyd1, which is similar to TasHyd1 (Albuquerque et al., 2004). Thus, the increase in *Paracoccidioides* in TC may elevate the disease risk for *B. thuringiensis* cotton. However, *Trichoderma* and *Verticillium* produce bisorbicillinoids that react synergistically with one another and also increase the disease risk of plants (Abe et al., 2000; Harned and Volp, 2011). Interestingly, the abundance of *Paracoccidioides* displayed a trend that was similar to *Verticillium* in different soil types. This finding suggests that *Paracoccidioides* possess a considerable capacity for plant root attachment and cooperative activity with *Verticillium.* Furthermore, *Paracoccidioides brasiliensis*, a human pathogenic fungus, is an etiological agent of paracoccidioidomycosis (Franco, 1987; Felipe et al., 2005). Recent publications provide very little information regarding the functions of *Poitrasia* (*P* < 0.002 for CC vs. TC-10 and for TC-10 vs. TC-15mix in both regions) and *Strobiloscypha*. The abundance of *Sporobolomyces*, a type of phyllosphere fungi, was lowest in TC-15mix during all three sampling stages (*P* = 0.001 for CC vs. TC-15mix). This fungi is beneficial to plants and antagonistic to *Cochliobolus sativus, Septaria nodorum* and *Penicillium expansum*, which cause blue mold and storage decay in fruits (Fokkema and Van der Meulen, 1976; Bashi and Fokkema, 1977). The decrease in *Sporobolomyces* in TC-15mix could result in an increase in some pathogenic fungi such as *Paracoccidioides* and *Verticillium*. Regarding the fungi *Alternaria* and *Chaetomium*, a line through the point appeared in TC-10. *Alternaria* species are responsible for at least ten types of plant diseases (Acland et al., 1998; van Wees et al., 2003), and *Chaetomium* reverses the effects of some fungal pathogens such as *Venturia inaequalis* and *Fusarium oxysporum* (Di Pietro et al., 1992; Lee and Hanlin, 1999). Because *Chaetomium* was present in lower amounts in TC-10, a higher abundance of pathogenic *Pestalosphaeria* was detected. A comprehensive view of the patterns of behavior in the indicator taxa in the four sampling seasons revealed almost equivalent tendencies in the variation patterns for each corresponding season. The relative abundance of the communities recovered using each region revealed almost the same dynamic change in CC, TC-10, and TC-15mix at the higher taxonomic level (*Geomyces* and *Poitrasia* in **Figure 3**). These findings revealed the actual variation tendency in the corresponding taxa, with the exception of some individual trends that might be associated with the growth characteristics of a particular taxon. Although changes in the relative abundance of fungi in soil are complex, potential indicators with differences in abundance between conventional and transgenic cottons are valuable for assessing plant-induced perturbations.

## Conclusion

This study for the first time provided fungal diversity associated with *B. thuringiensis* cottons planted for more than 10 years based on 18S and ITS regions, and monitored the variation in fungal communities over an annual cycle of cotton growth. The diversity indexes and grouping patterns revealed no obvious differences between CC and TC-10, suggesting monoculture of one transgenic cotton cultivar had no effect on fungal diversity in comparison with conventional cotton. However, TC-15mix planting with various transgenic cottons implied dissimilar fungal diversities to those in CC and TC-10, especially in region II. This suggested that variations of microbial diversity may exist among different transgenic cultivars or lines, and the unintended variations between transgenic and conventional cottons may fall into the generally acceptable range. Also, fungal lineages obtained by amplified regions influenced the biodiversity evaluation. Thus further research should devote particular attention to variations among genetic modifications within GMPs and amplified regions. Meanwhile informative indicators might be important for monitoring their local environments.

## Author Contributions

Conceived and designed the experiments: GZ and BL. Performed the experiments: XQ, QS, and BZ. Analyzed the data: XQ, QS, YB, and HW. Wrote the paper: LD and XQ. Revised and approved the final version of the paper: GZ and LD.

## Conflict of Interest Statement

The authors declare that the research was conducted in the absence of any commercial or financial relationships that could be construed as a potential conflict of interest.

## Supplementary Material

FIGURE S1 **Rarefaction curves for the estimated OTU richness across sampling sites **(A)** and sampling seasons **(B)** in the two regions.** CC: samples collected from soil amended with conventional cotton. TC-10: samples collected from soil amended with transgenic cotton for 10 years. TC-15mix: samples collected from soil amended with various transgenic cottons for 15 years. S, seeding stage; B, bud stage; Bl, blooming stage; Bo, boll opening stage.

FIGURE S2 **Cluster analysis based on the UniFrac distance of soil samples at a 3% cut-off level. (A)** Phylogenetic tree; **(B)** Phylogenetic tree displayed using a circular display. The dot lines showed the similarity cutoff levels to cluster the soil samples into several main groups in **(A)**. In order to exhibit the cluster pattern more clearly, **(A)** was performed as the circle format of **(B)**. The details about sample number were shown in Supplementary Table S1. TC-15mix clustered more closely in the branches of Groups II and III, and Group IV contained TC-15mix samples excluding only one from CC in region I. In Region II CC and TC-10 clustered together in Groups I and III, and TC-15mix were separate from CC.
