# Supplementary figures and images for: Assessing Fungal Population in Soil Planted with Cry1Ac and CPTI Transgenic Cotton and Its Conventional Parental Line Using 18S and ITS rDNA Sequences over Four Seasons
Source: Front Plant Sci. 2016 Jul 12;7:1023. doi: 10.3389/fpls.2016.01023 (PMC4940383; doi:10.3389/fpls.2016.01023)

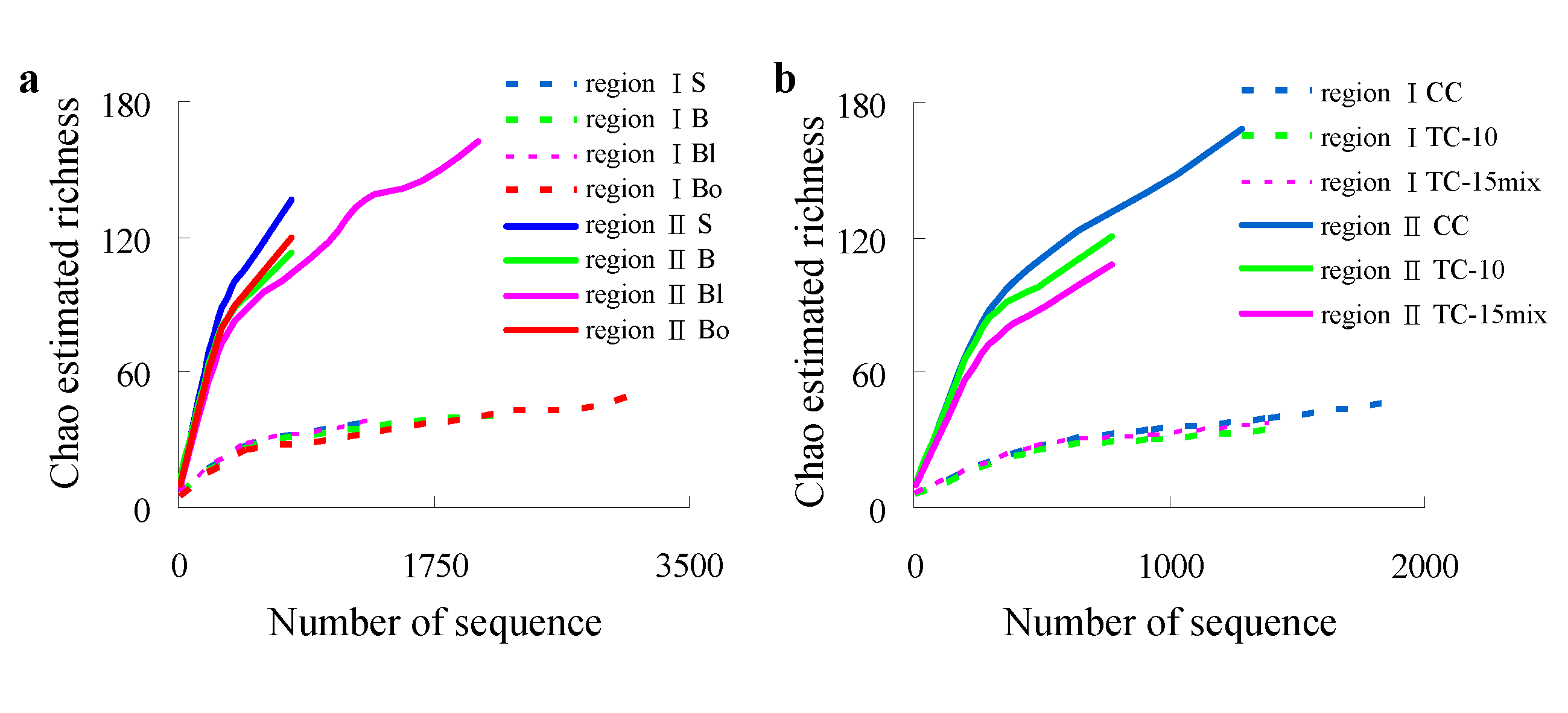

Supplement: FIGURE S1 — Rarefaction curves for the estimated OTU richness across sampling sites (A) and sampling seasons (B) in the two regions. CC: samples collected from soil amended with conventional cotton. TC-10: samples collected from soil amended with transgenic cotton for 10 years. TC-15mix: samples collected from soil amended with various transgenic cottons for 15 years. S, seeding stage; B, bud stage; Bl, blooming stage; Bo, boll opening stage. [file Image_1.TIFF]

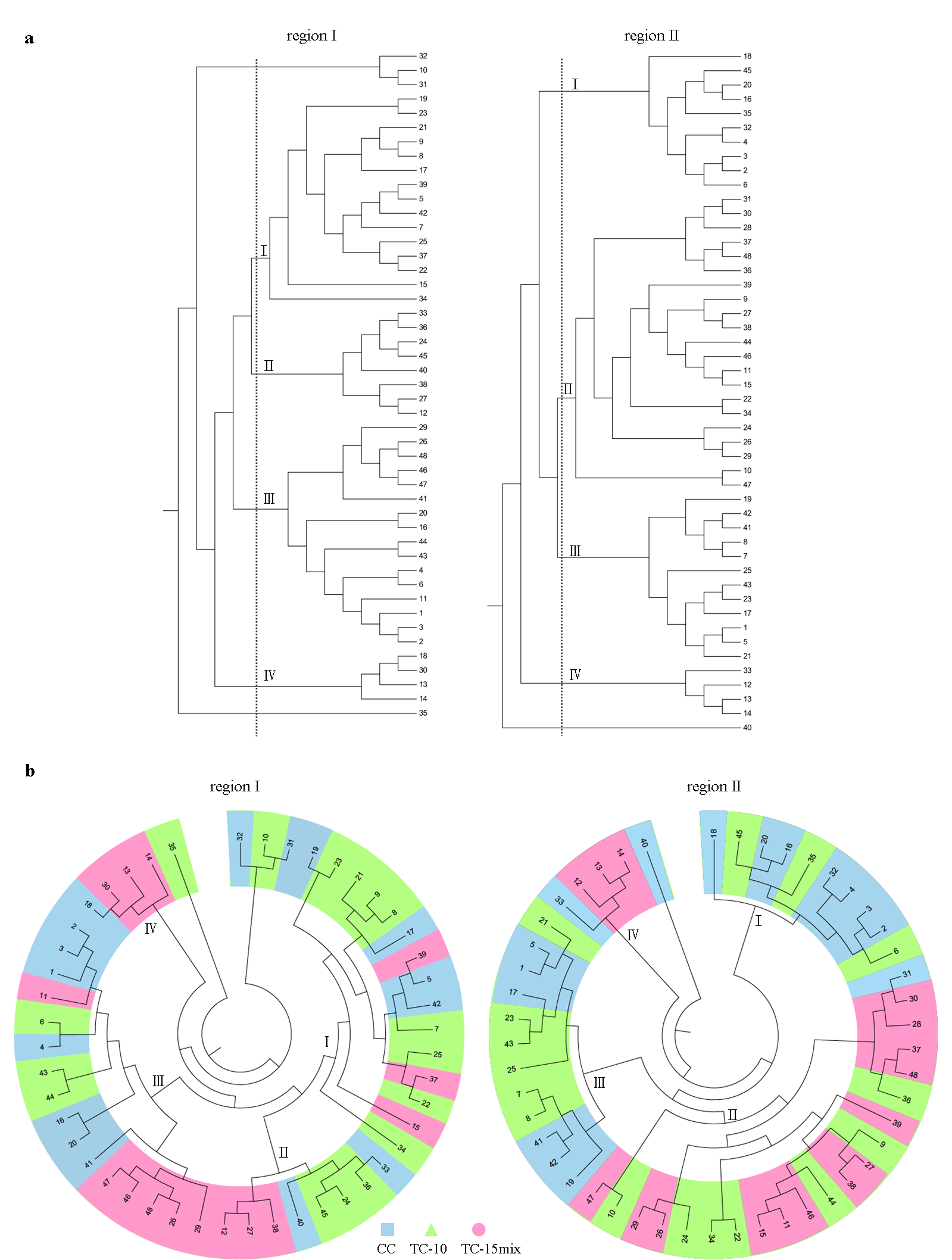

Supplement: FIGURE S2 — Cluster analysis based on the UniFrac distance of soil samples at a 3% cut-off level. (A) Phylogenetic tree; (B) Phylogenetic tree displayed using a circular display. The dot lines showed the similarity cutoff levels to cluster the soil samples into several main groups in (A). In order to exhibit the cluster pattern more clearly, (A) was performed as the circle format of (B). The details about sample number were shown in Supplementary Table S1. TC-15mix clustered more closely in the branches of Groups II and III, and Group IV contained TC-15mix samples excluding only one from CC in region I. In Region II CC and TC-10 clustered together in Groups I and III, and TC-15mix were separate from CC. [file Image_2.TIF]
