# Supplementary material for: Assessing Fungal Population in Soil Planted with Cry1Ac and CPTI Transgenic Cotton and Its Conventional Parental Line Using 18S and ITS rDNA Sequences over Four Seasons
Source: Front Plant Sci. 2016 Jul 12;7:1023. doi: 10.3389/fpls.2016.01023 (PMC4940383; doi:10.3389/fpls.2016.01023)
Supplement: Supplementary file 3 [file Table_1.DOC]

| **Supplementary Table S1. Characteristics and soil fertility variables of soil samples.** | | | | | | |
| --- | --- | --- | --- | --- | --- | --- |
| **Sampling site** | **Sample ID** | **Sampling time** | **TOC** | **TN** | **TP** | **TK** |
| CC | CC-S1 | S | 14.64 | 0.951 | 0.792 | 15.34 |
|  | CC-S2 | S | 14.73 | 0.997 | 0.811 | 15.94 |
|  | CC-S3 | S | 14.28 | 1.015 | 0.791 | 17.51 |
|  | CC-S4 | S | 15.30 | 0.922 | 0.859 | 16.07 |
|  | CC-S5 | S | 16.74 | 1.004 | 0.834 | 16.02 |
| TC-10 | TC-10-S1 | S | 18.69 | 1.019 | 0.866 | 14.68 |
|  | TC-10-S2 | S | 19.65 | 1.125 | 0.916 | 14.03 |
|  | TC-10-S3 | S | 17.76 | 1.032 | 0.837 | 13.94 |
|  | TC-10-S4 | S | 17.20 | 1.032 | 0.917 | 13.47 |
|  | TC-10-S5 | S | 15.04 | 0.955 | 0.875 | 15.08 |
| TC-15mix | TC-15-S1 | S | 14.63 | 1.030 | 0.856 | 16.40 |
|  | TC-15-S2 | S | 13.51 | 1.045 | 0.809 | 17.87 |
|  | TC-15-S3 | S | 15.62 | 1.049 | 0.770 | 14.18 |
|  | TC-15-S4 | S | 16.20 | 1.339 | 0.827 | 16.50 |
|  | TC-15-S5 | S | 13.94 | 1.022 | 0.815 | 15.74 |
| CC | CC-B1 | B | 16.29 | 1.240 | 0.759 | 16.10 |
|  | CC-B2 | B | 18.16 | 1.705 | 0.728 | 16.86 |
|  | CC-B3 | B | 13.21 | 1.098 | 0.744 | 14.26 |
|  | CC-B4 | B | 11.62 | 1.247 | 0.546 | 14.24 |
|  | CC-B5 | B | 14.32 | 1.059 | 0.733 | 13.89 |
| TC-10 | TC-10-B1 | B | 10.72 | 0.971 | 0.739 | 13.02 |
|  | TC-10-B2 | B | 20.64 | 1.040 | 0.701 | 14.03 |
|  | TC-10-B3 | B | 13.37 | 0.954 | 0.708 | 14.79 |
|  | TC-10-B4 | B | 15.55 | 2.201 | 0.739 | 16.01 |
|  | TC-10-B5 | B | 18.02 | 2.245 | 0.790 | 14.67 |
| TC-15mix | TC-15-B1 | B | 14.84 | 1.496 | 0.706 | 13.99 |
|  | TC-15-B2 | B | 12.01 | 1.377 | 0.769 | 15.78 |
|  | TC-15-B3 | B | 13.31 | 1.253 | 0.742 | 13.69 |
|  | TC-15-B4 | B | 14.89 | 1.361 | 0.734 | 14.01 |
|  | TC-15-B5 | B | 18.00 | 1.938 | 0.795 | 13.86 |
| CC | CC-Bl1 | Bl | 17.47 | 1.649 | 0.892 | 15.14 |
|  | CC-Bl2 | Bl | 15.83 | 1.329 | 0.703 | 13.05 |
|  | CC-Bl3 | Bl | 15.44 | 1.122 | 0.678 | 12.76 |
| TC-10 | TC-10-Bl1 | Bl | 15.77 | 1.042 | 0.859 | 14.37 |
|  | TC-10-Bl2 | Bl | 15.18 | 1.116 | 0.650 | 14.19 |
|  | TC-10-Bl3 | Bl | 13.23 | 1.237 | 0.908 | 16.76 |
| TC-15mix | TC-15-Bl1 | Bl | 14.03 | 1.237 | 0.801 | 13.12 |
|  | TC-15-Bl2 | Bl | 13.75 | 1.300 | 0.816 | 14.61 |
|  | TC-15-Bl3 | Bl | 13.82 | 0.948 | 0.778 | 15.06 |

| **Supplementary Table S1. Characteristics and soil fertility variables of soil samples (continued).** | | | | | | |
| --- | --- | --- | --- | --- | --- | --- |
| **Sample ID** | **Sampling site** | **Sampling time** | **TOC** | **TN** | **TP** | **TK** |
| CC | CC-Bo1 | Bo | 16.77 | 1.207 | 0.700 | 16.21 |
|  | CC-Bo2 | Bo | 18.55 | 1.189 | 0.671 | 14.82 |
|  | CC-Bo3 | Bo | 14.98 | 1.126 | 0.878 | 14.99 |
| TC-10 | TC-10-Bo1 | Bo | 15.38 | 1.515 | 0.800 | 14.41 |
|  | TC-10-Bo2 | Bo | 18.65 | 1.235 | 0.736 | 13.70 |
|  | TC-10-Bo3 | Bo | 14.49 | 1.024 | 0.625 | 11.53 |
| TC-15mix | TC-15-Bo1 | Bo | 12.24 | 1.311 | 0.959 | 15.28 |
|  | TC-15-Bo2 | Bo | 13.08 | 1.304 | 0.779 | 13.78 |
|  | TC-15-Bo3 | Bo | 11.45 | 1.015 | 0.711 | 13.45 |

All values are in g·kg^-1^.

TOC: total organic carbon. TN: total nitrogen. TP: total phosphorus. TK: total potassium.

S: seeding stage. B: bud stage. Bl: blooming stage. Bo: boll opening stage.
