# Supplementary material for: Assessing Fungal Population in Soil Planted with Cry1Ac and CPTI Transgenic Cotton and Its Conventional Parental Line Using 18S and ITS rDNA Sequences over Four Seasons
Source: Front Plant Sci. 2016 Jul 12;7:1023. doi: 10.3389/fpls.2016.01023 (PMC4940383; doi:10.3389/fpls.2016.01023)
Supplement: Supplementary file 4 [file Table_2.DOC]

| **Supplementary Table S2. Relative abundance (%) of fungal phyla across microbial communities of soil samples in region I.** | | | | | | |
| --- | --- | --- | --- | --- | --- | --- |
| **Sample ID** | **No.** | ***Ascomycota*** | ***Basidiomycota*** | ***Fungi_incertae_sedis*** | ***Glomeromycota*** | **Unclassified Fungi** |
| CC-S1 | 1 | 97 | 2.2 | 0 | 0 | 0.49 |
| CC-S2 | 2 | 98 | 0.99 | 0.015 | 0 | 0.95 |
| CC-S3 | 3 | 96 | 3.2 | 0 | 0 | 0.93 |
| CC-S4 | 4 | 98 | 0.20 | 0 | 0 | 2.0 |
| CC-S5 | 5 | 98 | 1.5 | 0.020 | 0.10 | 0.81 |
| TC-10-S1 | 6 | 98 | 0.038 | 0 | 0 | 1.6 |
| TC-10-S2 | 7 | 98 | 0.75 | 0.040 | 0 | 1.1 |
| TC-10-S3 | 8 | 99 | 0.42 | 0 | 0 | 0.46 |
| TC-10-S4 | 9 | 98 | 0.96 | 0 | 0 | 0.79 |
| TC-10-S5 | 10 | 77 | 0.98 | 0 | 0 | 22 |
| TC-15mix-S1 | 11 | 98 | 0.65 | 0.15 | 0.73 | 0.88 |
| TC-15mix-S2 | 12 | 93 | 0 | 0.39 | 0.039 | 7.0 |
| TC-15mix-S3 | 13 | 94 | 0 | 0.29 | 0.31 | 5.4 |
| TC-15mix-S4 | 14 | 91 | 0 | 0.71 | 1.0 | 7.3 |
| TC-15mix-S5 | 15 | 97 | 0.54 | 0.18 | 0.33 | 1.6 |
| CC-B1 | 16 | 90 | 7.2 | 0 | 0.025 | 2.8 |
| CC-B2 | 17 | 93 | 3.0 | 0.041 | 0.16 | 4.2 |
| CC-B3 | 18 | 92 | 1.8 | 0.024 | 0 | 6.2 |
| CC-B4 | 19 | 95 | 0.80 | 0 | 0.018 | 4.2 |
| CC-B5 | 20 | 92 | 3.51 | 0 | 0 | 4.5 |
| TC-10-B1 | 21 | 95 | 2.3 | 0.13 | 0 | 2.5 |
| TC-10-B2 | 22 | 96 | 1.4 | 0.024 | 0.024 | 2.7 |
| TC-10-B3 | 23 | 94 | 0.64 | 0 | 0 | 5.1 |
| TC-10-B4 | 24 | 97 | 0.30 | 0 | 0.013 | 2.2 |
| TC-10-B5 | 25 | 96 | 0.071 | 0 | 0 | 3.6 |
| TC-15mix-B1 | 26 | 95 | 0 | 0.38 | 0 | 4.7 |
| TC-15mix-B2 | 27 | 92 | 0 | 0 | 0.18 | 7.8 |
| TC-15mix-B3 | 28 | 96 | 0 | 0.21 | 0 | 3.4 |
| TC-15mix-B5 | 30 | 90 | 0.058 | 0.058 | 0 | 9.8 |
| CC-Bl1 | 31 | 81 | 1.6 | 0.060 | 0 | 17 |
| CC-Bl2 | 32 | 73 | 0.76 | 0.084 | 0 | 26 |
| CC-Bl3 | 33 | 93 | 0.84 | 0 | 0 | 6.3 |
| TC-10-Bl1 | 34 | 90 | 5.4 | 0 | 0.13 | 4.4 |
| TC-10-Bl2 | 35 | 76 | 21 | 0.089 | 0.30 | 2.6 |
| TC-10-Bl3 | 36 | 94 | 0.80 | 0 | 0.061 | 4.7 |
| TC-15mix-Bl1 | 37 | 95 | 0.90 | 0 | 0.060 | 4.3 |
| TC-15mix-Bl2 | 38 | 89 | 1.1 | 0 | 0.021 | 9.4 |
| TC-15mix-Bl3 | 39 | 99 | 0.19 | 0 | 0 | 1.3 |

| **Supplementary Table S2. Relative abundance (%) of fungal phyla across microbial communities of soil samples in Region I (continued).** | | | | | | |
| --- | --- | --- | --- | --- | --- | --- |
| **Sample ID** | **No.** | ***Ascomycota*** | ***Basidiomycota*** | ***Fungi_incertae_sedis*** | ***Glomeromycota*** | **Unclassified Fungi** |
| CC-Bo1 | 40 | 88 | 0.017 | 0.12 | 0 | 12 |
| CC-Bo2 | 41 | 96 | 0.87 | 0.015 | 0 | 3.2 |
| CC-Bo3 | 42 | 98 | 0.16 | 0.055 | 0.037 | 1.6 |
| TC-10-Bo1 | 43 | 96 | 0.35 | 0 | 0 | 3.2 |
| TC-10-Bo2 | 44 | 99 | 0.26 | 0.028 | 0 | 0.25 |
| TC-10-Bo3 | 45 | 97 | 0.14 | 0.060 | 0.040 | 2.7 |
| TC-15mix-Bo1 | 46 | 97 | 0 | 0.020 | 0 | 3.4 |
| TC-15mix-Bo2 | 47 | 97 | 0 | 0.12 | 0 | 2.9 |
| TC-15mix-Bo3 | 48 | 94 | 0 | 0.015 | 0 | 5.7 |

Frequency of the taxon in samples of different sampling sites was given as percentage, followed by the total number in all samples. Seasons were pooled, and the maximum number of samples was 47 in Region I (TC-15mix-B4 was discarded in downstream analysis for the sequence quality problem) and 48 in Region II. S: seeding stage; B: bud stage; Bl: blooming stage; Bo: boll opening stage.
