# Supplementary material for: Assessing Fungal Population in Soil Planted with Cry1Ac and CPTI Transgenic Cotton and Its Conventional Parental Line Using 18S and ITS rDNA Sequences over Four Seasons
Source: Front Plant Sci. 2016 Jul 12;7:1023. doi: 10.3389/fpls.2016.01023 (PMC4940383; doi:10.3389/fpls.2016.01023)
Supplement: Supplementary file 5 [file Table_3.DOC]

| **Supplementary Table S3. Relative abundance (%) of fungal phyla across microbial communities of soil samples in region II.** | | | | | |
| --- | --- | --- | --- | --- | --- |
| **Sample ID** | **Ascomycota** | **Basidiomycota** | **Fungi_incertae_sedis** | **Glomeromycota** | **Unclassified Fungi** |
| CC-S1 | 57 | 2.5 | 3.7 | 2.1 | 35 |
| CC-S2 | 49 | 1.6 | 4.7 | 0.85 | 44 |
| CC-S3 | 43 | 3.1 | 5.79 | 1.1 | 47 |
| CC-S4 | 38 | 1.1 | 8.4 | 7.4 | 45 |
| CC-S5 | 58 | 1.4 | 5.2 | 0.61 | 35 |
| TC-10-S1 | 49 | 0.047 | 4.0 | 0.75 | 47 |
| TC-10-S2 | 57 | 0.79 | 5.1 | 6.9 | 31 |
| TC-10-S3 | 56 | 0.50 | 3.1 | 1.3 | 39 |
| TC-10-S4 | 68 | 1.3 | 4.2 | 0.43 | 26 |
| TC-10-S5 | 51 | 1.4 | 24 | 2.5 | 21 |
| TC-15mix-S1 | 67 | 0.53 | 9.3 | 2.5 | 20 |
| TC-15mix-S2 | 63 | 0 | 28 | 0.51 | 8.9 |
| TC-15mix-S3 | 64 | 0 | 28 | 0.65 | 7.0 |
| TC-15mix-S4 | 67 | 0 | 19 | 2.1 | 12 |
| TC-15mix-S5 | 64 | 0.36 | 11 | 1.4 | 23 |
| CC-B1 | 33 | 5.2 | 13 | 0.99 | 49 |
| CC-B2 | 52 | 1.5 | 7.2 | 2.2 | 37 |
| CC-B3 | 40 | 1.9 | 24 | 0.36 | 33 |
| CC-B4 | 54 | 1.2 | 14 | 1.0 | 30 |
| CC-B5 | 29 | 2.3 | 25 | 1.4 | 43 |
| TC-10-B1 | 51 | 2.4 | 7.5 | 4.5 | 34 |
| TC-10-B2 | 64 | 1.3 | 7.9 | 1.0 | 25 |
| TC-10-B3 | 48 | 0.72 | 9.1 | 1.3 | 41 |
| TC-10-B4 | 62 | 0.50 | 6.5 | 2.0 | 29 |
| TC-10-B5 | 48 | 1.0 | 12 | 6.2 | 33 |
| TC-15mix-B1 | 58 | 0 | 6.5 | 3.9 | 32 |
| TC-15mix-B2 | 61 | 0 | 9.5 | 2.4 | 27 |
| TC-15mix-B3 | 52 | 0 | 15 | 1.1 | 31 |
| TC-15mix-B4 | 53 | 0.38 | 8.3 | 1.9 | 36 |
| TC-15mix-B5 | 54 | 0 | 8.2 | 1.4 | 36 |
| CC-Bl1 | 55 | 0.20 | 9.6 | 1.3 | 34 |
| CC-Bl2 | 38 | 2.0 | 14 | 1.2 | 46 |
| CC-Bl3 | 72 | 0.93 | 6.9 | 0.69 | 19 |
| TC-10-Bl1 | 60 | 6.5 | 11 | 2.3 | 21 |
| TC-10-Bl2 | 34 | 17 | 3.3 | 3.1 | 43 |
| TC-10-Bl3 | 56 | 1.9 | 16 | 1.0 | 25 |
| TC-15mix-Bl1 | 61 | 0.86 | 3.8 | 2.1 | 33 |
| TC-15mix-Bl2 | 62 | 1.3 | 9.5 | 1.8 | 26 |
| TC-15mix-Bl3 | 77 | 0.55 | 4.3 | 1.3 | 17 |

| **Supplementary Table S3. Relative abundance (%) of fungal phyla across microbial communities of soil samples in Region II (continued).** | | | | | |
| --- | --- | --- | --- | --- | --- |
| **Sample ID** | **Ascomycota** | **Basidiomycota** | **Fungi_incertae_sedis** | **Glomeromycota** | **Unclassified Fungi** |
| CC-Bo1 | 14 | 0.68 | 71 | 1.2 | 14 |
| CC-Bo2 | 59 | 2.2 | 6.6 | 1.2 | 31 |
| CC-Bo3 | 59 | 0.35 | 9.9 | 1.9 | 29 |
| TC-10-Bo1 | 50 | 0.43 | 5.7 | 1.5 | 42 |
| TC-10-Bo2 | 67 | 0.50 | 2.6 | 2.0 | 27 |
| TC-10-Bo3 | 46 | 0.61 | 2.7 | 0.33 | 50 |
| TC-15mix-Bo1 | 71 | 0 | 5.2 | 1.1 | 23 |
| TC-15mix-Bo2 | 53 | 0.29 | 7.8 | 13 | 27 |
| TC-15mix-Bo3 | 57 | 0.13 | 6.2 | 1.1 | 36 |
