# Supplementary material for: Assessing Fungal Population in Soil Planted with Cry1Ac and CPTI Transgenic Cotton and Its Conventional Parental Line Using 18S and ITS rDNA Sequences over Four Seasons
Source: Front Plant Sci. 2016 Jul 12;7:1023. doi: 10.3389/fpls.2016.01023 (PMC4940383; doi:10.3389/fpls.2016.01023)
Supplement: Supplementary file 6 [file Table_4.DOC]

| **Supplementary Table S4. Relative abundance (%, mean ± SD) of fungal lineages in overall communities and in different groups of soil samples in region I.** | | | | | | |
| --- | --- | --- | --- | --- | --- | --- |
| **Sample type** | ***Ascomycota*** | ***Basidiomycota*** | ***Fungi_incertae_sedis*** | ***Glomeromycota*** | **Unclassified Fungi** | **Others** |
| CC-S (n=5) | 58 ± 8.6 | 0.0045 ± 0.0063 | 0.98 ± 0.65 | 0.013 ± 0.029 | 0.52 ± 0.22 | 41 ± 8.7 |
| TC-10-S (n=5) | 64 ± 6.3 | 0.0056 ± 0.013 | 0.45 ± 0.30 | 0 | 3.6 ± 7.0 | 32 ± 7.1 |
| TC-15mix-S (n=5) | 71 ± 6.7 | 0.25 ± 0.15 | 0.17 ± 0.23 | 0.35 ± 0.26 | 3.2 ± 2.4 | 25 ± 7.3 |
| CC-B (n=5) | 52 ± 12 | 0.01 ± 0.01 | 1.6 ± 1.0 | 0.022 ± 0.036 | 2.1 ± 1.1 | 44 ± 12 |
| TC-10-B (n=5) | 63 ± 7.5 | 0.019 ± 0.034 | 0.60 ± 0.55 | 0.0051 ± 0.0072 | 2.0 ± 0.66 | 35 ± 7.4 |
| TC-15mix-B (n=4) | 67 ± 5.2 | 0.11 ± 0.12 | 0.010 ± 0.019 | 0.034 ± 0.068 | 4.5 ± 2.0 | 28 ± 4.6 |
| CC-Bl (n=3) | 53 ± 6.4 | 0.031 ± 0.029 | 0.67 ± 0.25 | 0 | 5.2 ± 3.0 | 41 ± 5.3 |
| TC-10-Bl (n=3) | 59 ± 14 | 0.016 ± 0.028 | 5.4 ± 5.5 | 0.10 ± 0.063 | 2.3 ± 1.1 | 34 ± 23 |
| TC-15mix-Bl (n=3) | 68 ± 12 | 0 | 0.50 ± 0.31 | 0.018 ± 0.020 | 3.2 ± 2.4 | 29 ± 9.6 |
| CC-Bo (n=3) | 73 ± 8.7 | 0.047 ± 0.036 | 0.28 ± 0.37 | 0.010 ± 0.017 | 4.1 ± 4.7 | 22 ± 4.2 |
| TC-10-Bo (n=3) | 64 ± 16 | 0.017 ± 0.016 | 0.17 ± 0.088 | 0.0066 ± 0.011 | 1.2 ± 0.97 | 35 ± 15 |
| TC-15mix-Bo (n=3) | 71 ± 6.5 | 0.035 ± 0.037 | 0 | 0 | 2.9 ± 1.0 | 27 ± 6.7 |

Others include Alveolata, Metazoa, Chlorophyta, Cryptophyta, Streptophyta, stramenopiles, Unclassified_eukaryota.
