# Supplementary material for: Assessing Fungal Population in Soil Planted with Cry1Ac and CPTI Transgenic Cotton and Its Conventional Parental Line Using 18S and ITS rDNA Sequences over Four Seasons
Source: Front Plant Sci. 2016 Jul 12;7:1023. doi: 10.3389/fpls.2016.01023 (PMC4940383; doi:10.3389/fpls.2016.01023)
Supplement: Supplementary file 7 [file Table_5.DOC]

| **Supplementary Table S5. Relative abundance (%, mean ± SD) of fungal lineages in overall communities and in different groups of soil samples in region II.** | | | | | | |
| --- | --- | --- | --- | --- | --- | --- |
| **Sample type** | **Ascomycota** | **Basidiomycota** | **Fungi_incertae_sedis** | **Glomeromycota** | **Unclassified Fungi** | **Others** |
| CC-S (n=5) | 35 ± 7.1 | 4.0 ± 1.3 | 1.4 ± 0.61 | 1.6 ± 1.9 | 29 ± 5.6 | 29 ± 7.4 |
| TC-10-S (n=5) | 39 ± 9.3 | 5.5 ± 6.1 | 0.58 ± 0.42 | 1.6 ± 1.7 | 22 ± 5.9 | 31 ± 8.2 |
| TC-15mix-S (n=5) | 47 ± 3.9 | 14 ± 7.1 | 0.12 ± 0.17 | 1.0 ± 0.64 | 10 ± 4.5 | 28 ± 5.1 |
| CC-B (n=5) | 26 ± 7.8 | 10 ± 5.1 | 1.5 ± 0.86 | 0.73 ± 0.38 | 24 ± 3.1 | 38 ± 4.9 |
| TC-10-B (n=5) | 35 ± 7.4 | 5.4 ± 1.0 | 0.77 ± 0.50 | 1.9 ± 1.3 | 20 ± 2.6 | 36 ± 4.8 |
| TC-15mix-B (n=4) | 28 ± 9.3 | 5.0 ± 2.9 | 0.038 ± 0.085 | 0.93 ± 0.30 | 16 ± 5.6 | 50 ± 17 |
| CC-Bl (n=3) | 0.20 ± 0.21 | 0.012 ± 0.011 | 0.0092 ± 0.016 | 0.77 ± 0.63 | 1.2 ± 0.65 | 34 ± 8.6 |
| TC-10-Bl (n=3) | 28 ± 9.5 | 5.7 ± 3.7 | 4.5 ± 3.8 | 1.2 ± 0.52 | 16 ± 4.9 | 45 ± 4.6 |
| TC-15mix-Bl (n=3) | 39 ± 15 | 3.1 ± 0.74 | 0.48 ± 0.080 | 1.0 ± 0.28 | 14 ± 5.4 | 42 ± 16 |
| CC-Bo (n=3) | 22 ± 11 | 20 ± 28 | 0.63 ± 0.57 | 0.76 ± 0.11 | 13 ± 3.9 | 45 ± 18 |
| TC-10-Bo (n=3) | 32 ± 6.6 | 2.1 ± 0.78 | 0.31 ± 0.11 | 0.72 ± 0.46 | 24 ± 9.8 | 40 ± 9.5 |
| TC-15mix-Bo (n=3) | 21 ± 6.0 | 2.3 ± 1.1 | 0.053 ± 0.067 | 2.1 ± 3.1 | 9.5 ± 2.2 | 65 ± 10 |

Others include Cryptophyta, Haptophyceae, Stramenopiles, Alveolata, Apicomplexa, Intramacronucleata, Centramoebida, Euglenozoa, Annelida, Arthropoda, Chordata, Nematoda, Platyhelminthes, Porifera, Chlorophyta, Streptophyta, Cryptophyta, Unclassified_eukaryota.
