# Supplementary material for: Assessing Fungal Population in Soil Planted with Cry1Ac and CPTI Transgenic Cotton and Its Conventional Parental Line Using 18S and ITS rDNA Sequences over Four Seasons
Source: Front Plant Sci. 2016 Jul 12;7:1023. doi: 10.3389/fpls.2016.01023 (PMC4940383; doi:10.3389/fpls.2016.01023)
Supplement: Supplementary file 8 [file Table_6.DOC]

| **Supplementary Table S6. Result of the t-test on fungal OTU richness and Shannon diversity in there treatment fields.** | | | | | | |
| --- | --- | --- | --- | --- | --- | --- |
|  | **Index** | **Treatment** | ***n*** | **Mean** | ***t*** | ***P*** |
| region I | OTU richness | CC | 16 | 38.63 |  |  |
|  |  | TC-10 | 15 | 35.50 | 1.78 | 0.08 |
|  |  | TC-15mix | 16 | 36.40 | 1.52 | 0.14 |
|  | Shannon | CC | 16 | 2.50 |  |  |
|  |  | TC-10 | 15 | 2.36 | 1.29 | 0.21 |
|  |  | TC-15mix | 16 | 2.70 | 1.12 | 0.27 |
| region II | OTU richness | CC | 16 | 127.69 |  |  |
|  |  | TC-10 | 16 | 110.94 | 1.22 | 0.23 |
|  |  | TC-15mix | 16 | 95.00 | -0.31 | 0.76 |
|  | Shannon | CC | 16 | 3.88 |  |  |
|  |  | TC-10 | 16 | 3.69 | 0.93 | 0.36 |
|  |  | TC-15mix | 16 | 3.52 | -2.15 | 0.04 |

The *P* and t values between CC *vs*. TC-10, and TC-10 *vs*. TC-15mix were shown.
