# Supplementary material for: Assessing Fungal Population in Soil Planted with Cry1Ac and CPTI Transgenic Cotton and Its Conventional Parental Line Using 18S and ITS rDNA Sequences over Four Seasons
Source: Front Plant Sci. 2016 Jul 12;7:1023. doi: 10.3389/fpls.2016.01023 (PMC4940383; doi:10.3389/fpls.2016.01023)
Supplement: Supplementary file 9 [file Table_7.DOC]

| **Supplementary Table S7. Pearson’s correlation coefficients between OTU richness, shannon, and soil fertility variables (N = 47 for Region I and N = 48 for Region II, **P* < 0.05, ***P* < 0.01).** | | | | | | | |
| --- | --- | --- | --- | --- | --- | --- | --- |
|  |  | **OTU richness** | **Shannon** | **TOC** | **TN** | **TP** | **TK** |
| Region I | OTU richness | 1 | 0.31* | 0.01 | 0.01 | -0.07 | 0.19 |
|  | Shannon |  | 1 | -0.05 | -0.04 | 0.10 | 0.14 |
|  | TOC |  |  | 1 | 0.24 | 0.13 | -0.02 |
|  | TN |  |  |  | 1 | -0.06 | 0.06 |
|  | TP |  |  |  |  | 1 | 0.34* |
|  | TK |  |  |  |  |  | 1 |
| Region II | OTU richness | 1 | 0.38** | 0.26 | -0.19 | 0.04 | 0.07 |
|  | Shannon |  | 1 | -0.02 | 0.11 | -0.08 | 0.08 |
|  | TOC |  |  | 1 | 0.23 | 0.14 | -0.01 |
|  | TN |  |  |  | 1 | -0.06 | 0.05 |
|  | TP |  |  |  |  | 1 | 0.35* |
|  | TK |  |  |  |  |  | 1 |
